# The Role of Bradykinin Receptors in the Etiopathogenesis of Chronic Spontaneous Urticaria

**DOI:** 10.3390/medicina57101133

**Published:** 2021-10-19

**Authors:** Aleksander Obtulowicz, Pawel Dubiela, Wojciech Dyga, Kamila Migacz-Gruszka, Tomasz Mikolajczyk, Anna Wojas-Pelc, Krystyna Obtulowicz

**Affiliations:** 1 Department of Dermatology, Jagiellonian University Medical College, Kopernika 50, 31-501 Krakow, Poland; obtulowicz@poczta.fm (A.O.); kamila.migacz@gmail.com (K.M.-G.); wojaspelca@su.krakow.pl (A.W.-P.); 2 Department of Pathophysiology and Allergy Research, Medical University of Vienna, Waehringer Guertel 18-20, A-1090 Vienna, Austria; 3 Department of Regenerative Medicine and Immune Regulation, Medical University of Bialystok, 15-269 Bialystok, Poland; 4 Department of Clinical and Environmental Allergology, Jagiellonian University Medical College, Botaniczna 3, 31-503 Krakow, Poland; wojciech.dyga@uj.edu.pl (W.D.); po80@wp.pl (K.O.); 5 Department of Internal and Agricultural Medicine, Jagiellonian University Medical College, Skarbowa 1, 31-121 Krakow, Poland; tomaszp.mikolajczyk@uj.edu.pl

**Keywords:** angioedema, bradykinin, bradykinin receptors, dermatology, urticaria

## Abstract

*Background and Objectives:* Chronic spontaneous urticaria (CSU) is a distressing skin condition, which manifests as red, swollen, itchy, and sometimes painful hives or wheals appearing on skin. Recently, CSU has been associated with bradykinin release, which was previously discovered to be the main trigger of hereditary angioedema attacks. To study the role of bradykinin receptors 1 (BR1) and 2 (BR2) in the etiopathogenesis of CSU. *Materials and Methods:* A total of 60 individuals, 30 patients with CSU and 30 healthy subjects, were recruited to the study. CSU was diagnosed in accordance with the standardized protocol of dermatological assessment of skin symptoms. The level of bradykinin receptors was determined in populations of CD3^+^, CD4^+^, and CD8^+^ lymphocytes as well as in CD14^++^CD16^−^, CD14^++^CD16^+^ and CD14^+^CD16^+^ monocytes. In addition, urticaria activity score summed over 7 days (UAS-7) was assessed and correlated with BR1 and BR2 expression. *Results:* A statistically significant higher concentration of BR1 expression in lymphocytes was found in patients with CSU, compared to the control group (*p* < 0.001). Moreover, a statistically significant positive correlation was observed between UAS-7 and BR1/BR2 expression in CD14^++^CD16^−^ cells (*p* = 0.03, R = 0.4). *Conclusions*: Bradykinin receptors are elevated in selected populations of lymphocytes in symptomatic CSU patients compared to healthy controls, indicating their role in the etiopathogenesis of the disease.

## 1. Introduction

Chronic spontaneous urticaria (CSU) is an inflammatory disease of complex etiopathogenesis of the dermatosis, hallmarked by the occurrence of at least 6 weeks of urticaria, which disappears spontaneously [1]. Despite numerous publications [2,3,4,5,6,7,8,9,10,11,12] indicating the role of humoral and cellular elements of the immunological system, coagulation factors and fibrinolysis, or the importance of a non-infective inflammatory component in its etiopathogenesis, the patomechanism of urticaria is still not fully understood [2]. So far, the role of histamine and IgE has been demonstrated, with clinical implications for treatment with histamine antagonist and omalizumab [1,13].

Angioedema is a common symptom of CSU and has a substantial effect on patients’ quality of life [1]. It is estimated that 33–67% of patients with CSU exhibit both hives and angioedema, 29–65% exhibit only hives, and 1–13% exhibit only angioedema [14,15]. Although for many years angioedema was believed to be exclusively mediated by histamine release in CSU patients, recent results from Hoffmann et al. indicate the role of bradykinin (BK), which was previously associated with another disease, namely, hereditary angioedema [16,17].

C1-inhibitor deficiency causes dysregulation of the plasma bradykinin-forming cascade with overproduction of bradykinin, due to the uninhibited effects of activated factor XII and plasma kallikrein, and thus triggers hereditary angioedema (HAE) [17]. BK, as a pro-inflammatory mediator in the periphery, can elicit all major signs of inflammation; specifically, pain, hyper-perfusion, and increased vascular permeability [7,18,19,20]. BK activates two types of receptors: the B1 receptor (bradykinin receptor 1 (BR1)), and the B2 receptor (bradykinin B receptor 2 (BR2)]) [8,9]. BR2 is a constitutive receptor and has high affinity for BK, while BR1 is generally upregulated following tissue injury and binds with high affinity to des-Arg9-BK, a kinin metabolite [9,10]. Bradykinin receptors were shown to be overexpressed in patients with HAE. Following its nature, BR2 was found to be a target in HAE treatment and its antagonist, icatibant, is used as an on demand treatment for angioedema attacks [21].

Interestingly, the clinical symptoms of histamine and bradykinin induced angioedema are indistinguishable, which is the most important factor causing delays in HAE diagnosis [22]. The difference between those two types of angioedema can undoubtedly be found in response to the treatment. Histamine-mediated angioedema will almost always respond rapidly to aggressive treatment (with antihistamine, steroid, and possibly epinephrine). In contrast, bradykinin-mediated angioedema remains resistant to standard anti-allergic drugs, with a tendency to progress slowly, over a few hours [23]. Since allergy is more common and better understood, there is more knowledge about the coincidence of histamine and bradykinin release than the triggers involved in the pathology of anaphylaxis [24]. Anaphylaxis induces an increase in the levels of many mediators (various cytokines and chemokines, prostaglandins, tryptase, bradykinin, serotonin) which could potentially contribute (positively or negatively) to the clinical signs and symptoms. Depletion of the bradykinin precursor, high molecular weight kininogen, has been observed in anaphylaxis, likely through activation of the plasma contact system and kallikrein [25]. On the other hand, nothing is known about the role of histamine in HAE attacks.

Regarding angioedema in CSU, the role of histamine is well-established. Triggered by the interplay of immunoglobulin E (IgE) and mast cells and basophils, it is a key mediator released from the cells [1]. The latest results indicate that BK could also be involved in the attacks involving swelling in CSU. Hoffmann et al. unambiguously demonstrated that cleaved high molecular weight kininogen (cHMWK) levels were elevated in symptomatic CSU patients, reflecting an increased bradykinin release in comparison to healthy controls. Interestingly, cHMWK levels were not limited to patients with angioedema [16]. Although the available data provide some insights regarding the potential role of BK in urticaria, the authors agreed that more studies are required to understand its clinical implications. Thus, this study was performed on a well-characterized cohort to determine whether bradykinin receptors are overexpressed in patients with CSU and if it corresponds with severity of the disease. 

## 2. Materials and Methods

### 2.1. Chemicals and Reagents

All reagents were purchased from Sigma-Aldrich (Saint Louis, MO, USA), unless stated otherwise.

### 2.2. Study Participants and Blood Collection

All participants from the center, who entered the study, were diagnosed with CSU in accordance with international guidelines (EAACI/GA) and LEN/EDF [1], and thus defined as having had spontaneous recurrent wheals and/or angioedema for at least 6 weeks in the period prior to the study. To avoid additional bias and determine if urticaria can be triggered by bradykinin we limited our cohort to patients suffering only from urticaria, and not from angioedema; this also eliminated the possibility of having impacts from causes of angioedema other than CSU. A total of 30 CSU patients (Male = 6, Female = 24; age 19–87, median 42.5) and 30 healthy controls with age (23–71, median 40.8) and sex rate (Male = 7, Female = 23) adapted to the study design, to eliminate any bias, entered the study. All the subjects underwent consultations (dental, ENT, urological, and gynecological in women) to exclude any unknown inflammation. Urticaria activity was assessed using the urticaria activity score summed over 7 days (UAS-7), in accordance with the EAACI/GA and LEN/EDF [1] guidelines. Importantly, neither patients nor healthy control subjects were treated with ACE-inhibitors when entering the study.

Blood samples were collected from the patients with active urticaria and healthy controls. Standard blood tests, including D-Dimer concentration, CRP, level of fibrinogen and vitamin D (excluding patients nr 12, 26, 29 with no data available), total IgE level, and number of thrombocytes, were performed in a certified laboratory.

The study was approved by the local Ethics Committee of Jagiellonian University in Cracow (104/B2014, 22 May 2014).) and conducted in accordance with the Declaration of Helsinki. Patients gave their written informed consent. All experiments were performed in accordance with the relevant guidelines and regulations. More detailed information about patients clinical characteristics is shown in Table 1.

### 2.3. Cell Staining and Flow Cytometry

Peripheral blood mononuclear cells (PBMCs) were isolated from the peripheral blood of the donors by density gradient separation with Ficoll-Hypaque (PAN BiotechGmbh, Aidenbach, Germany). Cells, after isolation, were washed three times in PBS (Phosphate buffered saline) (Gibco, Grand Island, NY, USA) with 1% FBS (Fetal bovine serum) and stained with monoclonal antibodies: anti-CD3 PerCP (Clone SK7, BD, San Jose, CA, USA), anti-CD4 APC (Clone RPA-T4, BD), anti-CD8 APC-H7 (Clone SK1, BD), anti-HLA-DR APC (Clone L243, BD), anti-CD14 PerCP (Clone MoP9, BD), and anti–CD16APC-H7 (Clone 3G8, BD).

To assess the expression of the BR1, cells were stained for 30 min at 4 °C with anti-BDKRB1 FITC (Polyclonal, Bioss Antibodies, Woburn, MA, USA). To assess the expression of the BR2, cells were first permeabilized for 30 min at 4 °C using a Fixation/Permeabilization set (eBioscience, San Diego, CA, USA) and stained for 30 min at 4 °C with rabbit monoclonal antibodies anti-BDKRB2 (Clone: EPR5646, Abcam, Cambridge, UK) combined with mouse monoclonal secondary antibody anti-rabbit IgG PE (Clone 2A9, Abcam).

Cells were analyzed using a BD FACSVerse^TM^ flow cytometer with BD FACSuite software (BD Biosciences) and FlowJo software (FlowJo, Ashland, OR). Cell viability was determined using the fixable viability dye eFluor 780 (eBioscience). A standardized gating strategy was employed. First, lymphocytes were gated in accordance with forward scatter/side scatter. Furthermore, we focused on the following cell types: T cells (CD3^+^), Th lymphocytes (CD3^+^ CD4^+^ CD8^−^), Tc lymphocytes (CD3^+^, CD8^+^, CD4^−^), monocytes (HLA-DR^+^CD14^+^), with their classical (HLA-DR^+^CD14^++^CD16^−^) and intermediate (HLA-DR^+^CD14^++^CD16^+^) subsets. All applied antibodies are listed in Table S1 (Supplementary Materials). Gating strategy is presented in the Supplementary Materials (Figure S1).

### 2.4. Statistical Analyses

All the analyses were performed with IBM SPSS Statistics 25. Data were presented as median and lower and upper quartiles (Q1; Q3). Box and whisker plots were generated with IBM SPSS Statistics 25 and showed the median, interquartile range (IQR), and minimum and maximum values. Normality of the data was assessed using a Shapiro–Wilk test. A Mann–Whitney test was used to compare patient and control groups, as well as to compare bradykinin-treated and untreated PBMCs. Spearman’s test was used to measure the strength of association between two variables. *p*-values of <0.05 were considered statistically significant.

## 3. Results

### 3.1. Comparison of Lymphocytes and Monocytes Subpopulations Distribution

A comparison of lymphocytes and monocytes subpopulation distribution between examined groups showed a significantly increased subpopulation of CD4^+^ (69.3; 64.5–75.0 vs. 58.3; 54.7–62.6, *p* < 0.0001) and decreased subpopulation of CD8^+^ (24.6; 20.2–29.3 vs. 33.3; 30.9–37.3) in the CSU patient group, compared to the healthy control (Table 2).

### 3.2. Expression of Bradykinin Receptors

BR1 expression in CD3^+^ cells was significantly higher (*p <* 0.001) in CSU patients (9.12; 7.17–16.78) in comparison to healthy controls (3.8; 3.08–5.20). The same statistically significant correlation (*p <* 0.001) was found for comparison of CSU vs. healthy controls median expression of BR1 on CD4^+^ (6.96; 5.78–10.57 vs. 2.90; 2.31–4.28) and CD8^+^ (12.84; 9.77–22.64 vs. 6.05; 4.51–8.95), respectively. Notably, a significantly higher (*p <* 0.001) median concentration of BR1 expression in CD8^+^ cells (12.84; 9.77–22.64) was found in comparison to CD4^+^ (6.96; 5.78–10.57) in patients with CSU. In addition, the expression of BR1 detected in monocytes HLA-DR^+^CD14^+^ in CSU patients (37.2; 31.3–42.4) was significantly higher (*p <* 0.001) in comparison to healthy controls (26.2; 22.1–30.9). A similar relationship was found, both in classic CD14^++^CD16^−^ (29.9; 25.2–37.7 vs. 19.8; 17.6–25.3), as well as intermediate CD14^++^CD16^+^ (54.6; 48.6–75.1 vs. 37.5; 33.4–54.1) subsets of monocytes. Among monocytes, a higher expression of BR1 on CD14^++^CD16^+^ (54.6; 48.6–75.1) was detected compared to CD14^++^CD16^−^ (29.9; 25.2–37.7) in CSU patients.

BR2 expression levels between the groups were comparable, and statistically significant differences were not found; neither for CD3^+^ cells (9.0; 4.0–14.2 vs. 7.7; 4.4–18.1) nor for CD4^+^ (9.7; 3.7–21.1 vs. 7.6; 4.4–14.9) and CD8^+^ (13; 10–22.7 vs. 12.2; 4.6–22.9). However, a higher expression of BR2 in monocytes in CSU patients was found for HLA-DR^+^CD14^+^ cells (80.3; 65.7–88.5 vs. 63.5; 56.3–78.9; *p* < 0.019) and their classic subset CD14^++^CD16^−^ (83.1; 73.9–89.2 vs. 68.6; 62.1–82.3; *p* < 0.003). All the results are presented in Table 3. Box plots and representative flow cytometric examples are presented in the Supplementary Materials (Figures S2–S5).

### 3.3. Disease Activity Correlation with BR1 and BR2

**Figure 1:**
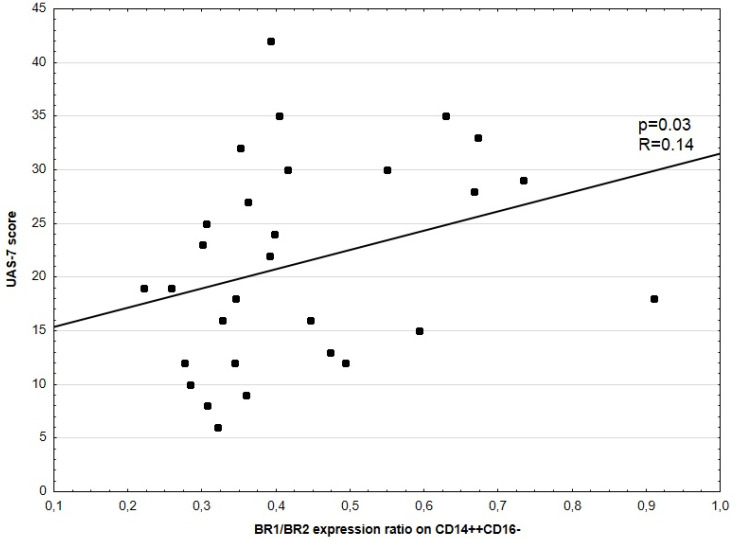
Correlation between UAS-7 score and BR1/BR2 expression ratio in CD14^++^CD16^−^ cells. Each dot represents one patient with their ID.

**Figure 2:**
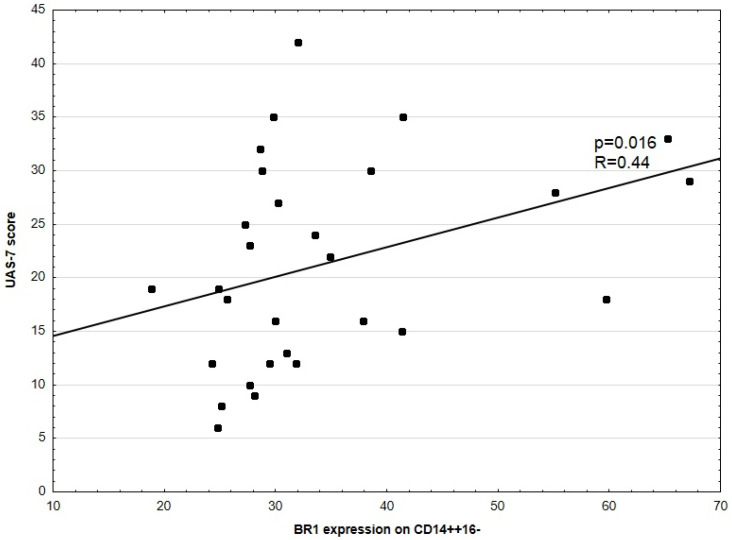
Correlation between UAS-7 score and BR1 expression in CD14^++^CD16^−^ cells. Each dot represents one patient.

**Figure 3:**
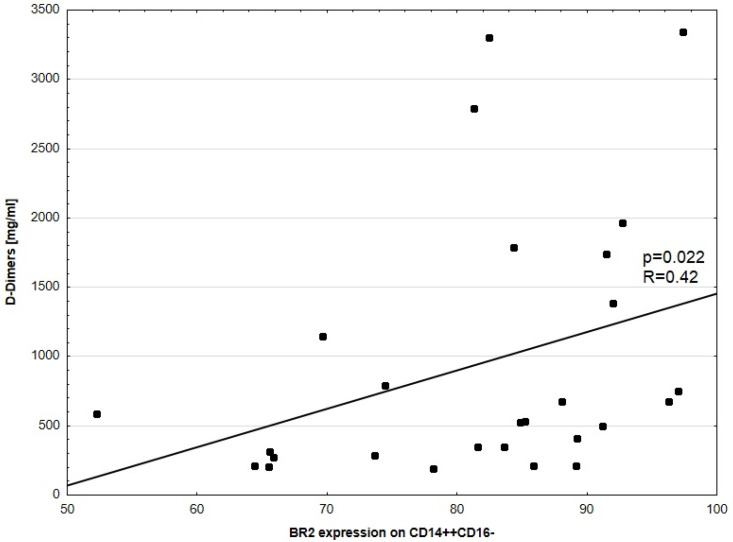
Correlation between D-Dimer concentration and BR2 expression in CD14^++^CD16^−^ cells. Each dot represents one patient.

No significant correlation between disease activity (UAS-7) and BR2 expression in CD14^++^16^–^ was noted. No significant correlations between UAS-7 and BR1, as well as BR2 expression in CD3^+^, CD4^+^, CD8^+^, HLA-DR^+^CD14^+^, and CD14^++^CD16^+^ were observed. There was a weakly positive, but statistically significant, correlation between disease activity (UAS-7) and BR1 to BR2 ratio expression in CD14^++^CD16^−^ (*p* = 0.03, R = 0.14, Figure 1). There was also a significant correlation between CSU activity and BR1 expression in CD14^++^CD16^−^ (*p* = 0.016, R = 0.44) and D-Dimers concentration and BR2 expression in CD14^++^CD16^−^ cells, respectively (Figure 2 and Figure 3).

### 3.4. D-Dimers Concentration and BR Expression

**Figure 4:**
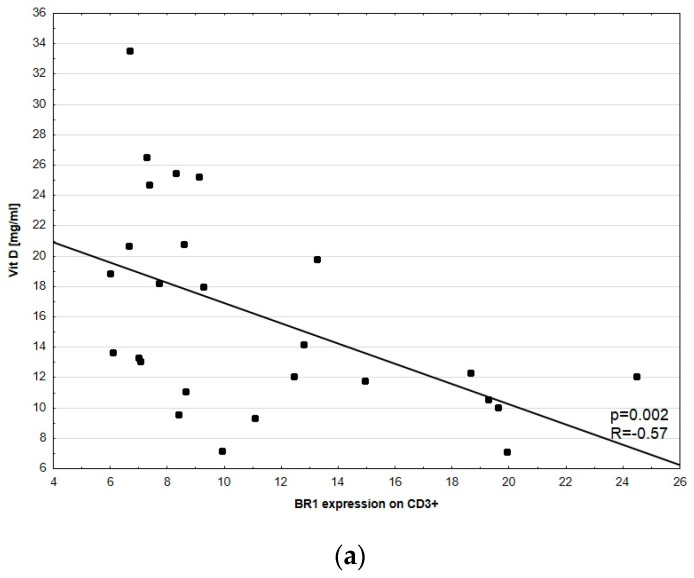

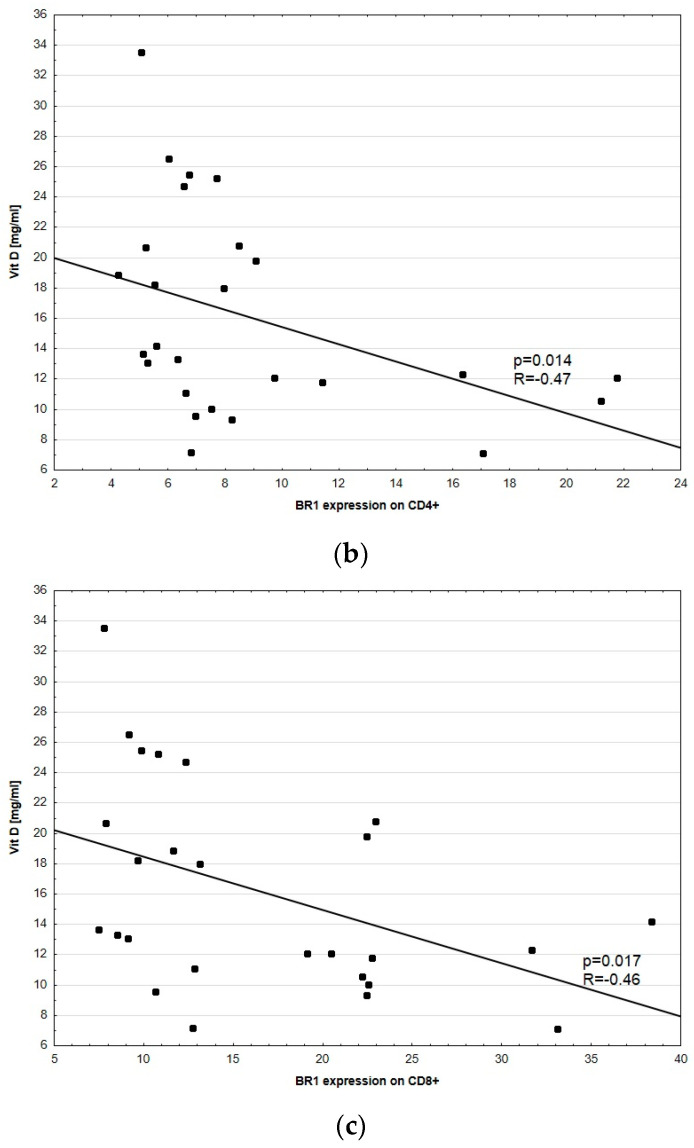
Correlation between vitamin D concentration and BR1 expression in (**a**) CD3^+^, (**b**) CD4^+^ and (**c**) CD8^+^ cells (excluding patients No. 12, 26, and 29; no results of vitamin D). Each dot represents one patient with their ID.

No significant correlations between D-dimer concentration and BR1 expression in CD3^+^, CD4^+^, CD8^+^, HLA-DR^+^CD14^+^, and CD14^++^CD16^+^ were observed. A significant correlation between D-dimer concentration and BR2 expression in CD3^+^, CD4^+^, CD8^+^, HLA-DR^+^CD14^+^, and CD14^++^CD16^+^ was not noted. However, we found a statistically significant negative correlation between vitamin D concentration and BR1 expression in all tested lymphocyte subsets, namely CD3^+^ (*p* = 0.002, R = −0.57), CD4^+^ (*p* = 0.014, R = −0.47), and CD8^+^ (*p* = 0.017, R = −0.46, Figure 4).

There were no statistically significant correlations between the other tested blood parameters: CRP, fibrinogen, IgE concentration, and thrombocyte levels and bradykinin receptors expression (Table 4).

## 4. Discussion

CSU is a complex disease with a major burden on patients and significant healthcare costs and socio-economic implications [26,27]. Treating-to-target, as in other chronic diseases, should be the goal of CSU management. The significant problem with the disease is an unclear mechanism, which causes difficulties in patient management. Since not all of patients respond to the standard treatment that covers histamine-mediated disease, we speculated that other mediators could be involved in triggering the symptoms. Bradykinin has similar molecular characteristics to histamine and was recently shown by Hoffmann et al. to be elevated in CSU patients [16]. Therefore, we aimed to show the role of bradykinin receptor expression in patients with CSU. From a clinical perspective, finding elevated bradykinin receptors levels is a critical point to investigate when deciding whether their antagonists can be considered for treatment. We tested the expression of BR1 and BR2 on selected subsets of lymphocytes and checked the correlation with blood parameters known to be involved, i.e., levels of vitamin D or D-dimers. In our study we measured an increased level of bradykinin receptors in patients with CSU. We also determined the correlation between BR1/BR2 ratio and UAS-7. Our novel study suggests that beside bradykinin, its receptors could also be involved in triggering urticaria symptoms.

There was substantial dysregulation between the CD4^+^ and CD8^+^ cell subpopulations in patients with CSU compared to healthy controls, favoring Th cells, as previously described [28]. Our initial experiment, performed to characterize cell subpopulations, confirmed the clinical characteristics and cohort selection on a cellular level. We further observed an increase of BR1 in the lymphocytes of CSU patients, in comparison to healthy controls. Notably, this was observed for CD3^+^, CD4^+^, and CD8^+^ cells. Similar observations were found for BR2 in tested cells. Interestingly, there was a significantly higher expression of BR1 in CD8^+^ than CD4^+^, which could confirm the results of previous studies showing that chronic non-infectious inflammation in the vascular endothelium plays a critical role in etiopathogenesis of the disease [19,29,30,31,32,33,34,35]. These observations are also in line with previous results in HAE patients [36,37], showing that BR1 in lymphocytes and BR2 in monocytes play a role, not only in HAE, but also in CSU. It is of great importance to consider previous studies that excluded the impact of bradykinin in CSU and HAE with a normal C1-inhibitor level [38,39,40,41].

The role of bradykinin receptor expression in the etiopathogenesis of CSU was also confirmed in experiments that aimed to correlate the severity of the disease measured by UAS-7, and BR1 and BR2 expression level in monocytes. Our analysis confirmed that this correlation is more dependent on BR1 in CD14^++^CD16^−^ monocytes, and the BR2 activity is combined with D-dimer concentration values. Considering previous studies [3,29] showing a correlation between CSU severity measured by UAS-7, and D-dimer level, we can speculate that the bradykinin receptors that we combined with previously examined parameters are involved in the disease progression. This hypothesis is supported by clinical observations showing that with increasing severity of CSU, patients become more resistant to the standard treatment with antihistamine [42]. Perhaps, there is another mediator involved in the process. The results from Hoffman et al., and ours, strongly imply the role of bradykinin.

Considering molecular mechanism of bradykinin in initiation of inflammation in the vascular endothelium mediated by BR1, it was previously shown that the receptor is eliciting expression of adhesion molecules on endothelial cells, which ultimately leads to uncontrolled development of the inflammatory reaction [30,31,32,33]. Our previous study showed that this process is also clinically associated with uncontrolled urticaria and elevated levels of D-Dimers in CSU patients [19,29] that was also observed in this study.

Considering the role of BR2, which was significantly correlated with D-dimer concentration and revealed opposite characteristics to BR1, we speculate that it could be a follow up mechanism. In the disease, after a reaction mediating the inflammation and increased severity, BR2 might be related to the protection of epithelial cells [3,43,44]. These results, in turn, show the modulatory effect of BR2 in inflammatory process in the endothelium. If we combine the proposed roles of BR1 and BR2, we find a sophisticated interplay that explains the alternating seizures and self-limitation of urticaria observed in the clinic [45].

Since there are several studies [20,46,47,48] showing a correlation between the level of vitamin D and CSU activity, we wanted to determine whether this also correlates with BR. A negative correlation with BR1 and positive with UAS-7 was found in the study. This, in turn, suggests that higher vitamin D levels are linked with faster disease progression and the occurrence of more severe symptoms, which is contradictory to previous reports. The vitamin D level is dependent on many factors and its correlation with disease severity can also be cohort dependent, as summarized in a review [49]. Since many CSU patients had significantly lower serum vitamin D levels than the controls in most studies, there were some trials performed to check the efficacy of its implementation. The results did not show the effect of the treatment unambiguously. Our results show that in the etiopathogenesis of CSU, in a cohort of patients with normal vitamin D concentration, bradykinin receptors are involved in triggering symptoms and are associated with UAS-7 score. More studies need to be performed to fully understand this mechanism.

The results of this research indicate that a BK involving coagulation system, fibrinolysis, and activation of the endothelium may be some of the missing players in understanding the CSU mechanism. This study supports results from Hoffmann et al. and opens up the discussion regarding new treatment approaches for patients with CSU that are resistant to antihistamine and omalizumab treatment. Bradykinin receptors, previously associated with HAE and tested in the treatment of recurrent angioedema attacks, can be a new molecular target for CSU patients for better patient management. However, a clinical study needs to be performed to prove our hypothesis and results obtained at a cellular level.

## Figures and Tables

**Table 1:** Patients’ characteristics and disease severity. Disease severity defined as: symptom-free = complete disease control, mild = symptoms partially controlled, no need for additional therapy (UAS-7 score 7–15), moderate to severe = uncontrolled symptoms, need for additional therapy (UAS-7 score 16–42).

**Number of CSU Patients**	**30**
Sex Male/Female n (%)	6 (20%)/24 (80%)
Mean age (range)	46.13 (+/−15.06)
**Disease Severity by UAS-7**	
Symptom-free n (%)	0 (0%)
Mild n (%)	9 (30%)
Moderate to severe n (%)	21 (70%)

**Table 2:** Cells characteristics in the two tested cohorts. Data are presented as median and lower and upper quartiles (Q1; Q3). Statistical significance of the results was analyzed with a Mann–Whitney test.

Subpopulation of Cells	Healthy Control Group Median (Q1–Q3) (%)	CSU Group Median (Q1–Q3) (%)	*p*-Value
**T lymphocytes** CD3^+^	61.4 (56.1–66.7)	65.9 (52.9–71.3)	<0.25
**Th** CD4^+^	58.3 (54.7–62.6)	69.3 (64.5–75.0)	<0.0001
**Tc** CD8^+^	33.3 (30.9–37.3)	24.6 (20.2–29.3)	<0.0001
**Monocytes** HLA-DR^+^CD14^+^	32.1 (21.5–38.8)	32.4 (21.6–46.0)	<0.73
**Monocytesintermediatesubset** CD14^++^CD16^+^	7.8 (4.8–13.0)	5.16 (3.5–8.9)	<0.09
**Monocytes Classicsubset** CD14^++^CD16^−^	86.0 (78.6–90.0)	89.6 (86.6–92.9)	<0.06

Abbreviation: CSU, chronic spontaneous urticaria.

**Table 3:** Median of bradykinin receptor 1 and 2 expression in CD3^+^, CD4^+^, and CD8^+^ cells. Statistical significance of the results was analyzed with a Mann–Whitney test.

	Healthy Control Group (% of Cells)	CSU Patients (% of Cells)	*p*-Value
**BR1 level on CD3^+^** (range)	3.8 (3.08–5.20)	9.12 (7.17–16.78)	<0.001
**BR1 level on CD4^+^** (range)	2.90 (2.31–4.28)	6.96 (5.78–10.57)	<0.001
**BR1 level on CD8^+^** (range)	6.05 (4.51–8.95)	12.84 (9.77–22.64)	<0.001
**BR2 level on CD3^+^** (range)	7.72 (4.44–18.06)	9.02 (3.97–14.23)	<0.773
**BR2 level on CD4^+^** (range)	7.63 (4.36–14.93)	9.72 (3.66–21.06)	<0.824
**BR2 level on CD8^+^** (range)	12.21 (4.58–22.94)	12.99 (9.96–22.68)	<0.957

Abbreviation: BR1/BR2—bradykinin receptor 1/2; CSU, chronic spontaneous urticaria.

**Table 4:** Correlation between tested blood parameters: CRP, fibrinogen, IgE concentration, thrombocyte levels, and bradykinin receptor expression in CSU patients. Statistical significance of the results was analyzed with a Spearman test.

	BR1 CD3^+^ *p*-Value	BR1 CD4^+^ *p*-Value	BR1 CD8^+^ *p*-Value	BR2 CD3^+^ *p*-Value	BR 2 CD4^+^ *p*-Value	BR 2 CD8^+^ *p*-Value
CRP	0.675	0.952	0.915	0.742	0.319	0.884
Fibrinogen	0.638	0.253	0.783	0.680	0.818	0.787
IgE level	0.701	0.147	0.808	0.830	0.660	0.889
Thrombocytes	0.081	0.312	0.074	0.795	0.799	0.914

## Data Availability

The data presented in this study are available on request from the corresponding author. The data are not publicly available due to privacy restrictions.

## Supplementary Materials

The following are available online at https://www.mdpi.com/article/10.3390/medicina57101133/s1. Figure S1: Gating strategy for the analysis of CD14^++^CD16^−^, CD14^++^CD16^+^, CD14^+^CD16^+^ monocytes subsets and CD4^+^ and CD8^+^ T cells subsets, respectively, Figure S2: Box plot of BR1 (A) and BR (2) expression on CD3^+^ (left panel) CD4^+^ (center panel) and CD8^+^ (right panel) cells, Figure S3: Box plot of BR1 (A) and BR (2) expression on Monocytes and their CD14^++^CD16^−^, CD14^++^CD16^+^, and CD14^+^CD16^+^ subsets, Figure S4: Representative dot plots for BR1 and BR2 expression on T cells **(**(right panel and FMO Control (left panel), Figure S5: Representative histograms for BR1 and BR2 expression on monocytes ((right panel and FMO Control (left panel)), Table S1: List of the antibodies used for the staining.
