# Supplementary material for: The Role of Bradykinin Receptors in the Etiopathogenesis of Chronic Spontaneous Urticaria
Source: Medicina (Kaunas). 2021 Oct 19;57(10):1133. doi: 10.3390/medicina57101133 (PMC8539896; doi:10.3390/medicina57101133)
Supplement: Supplementary file 1 [file medicina-57-01133-s001.zip › medicina-1401460-SI.pdf]

Supplementary Materials

Fig S1. Gating strategy for the analysis of CD14^{++}CD16^{-}, CD14^{++}CD16^{+}, CD14^{+}CD16^{+} and CD4+ and CD8+ monocytes and T cells subsets, respectively.

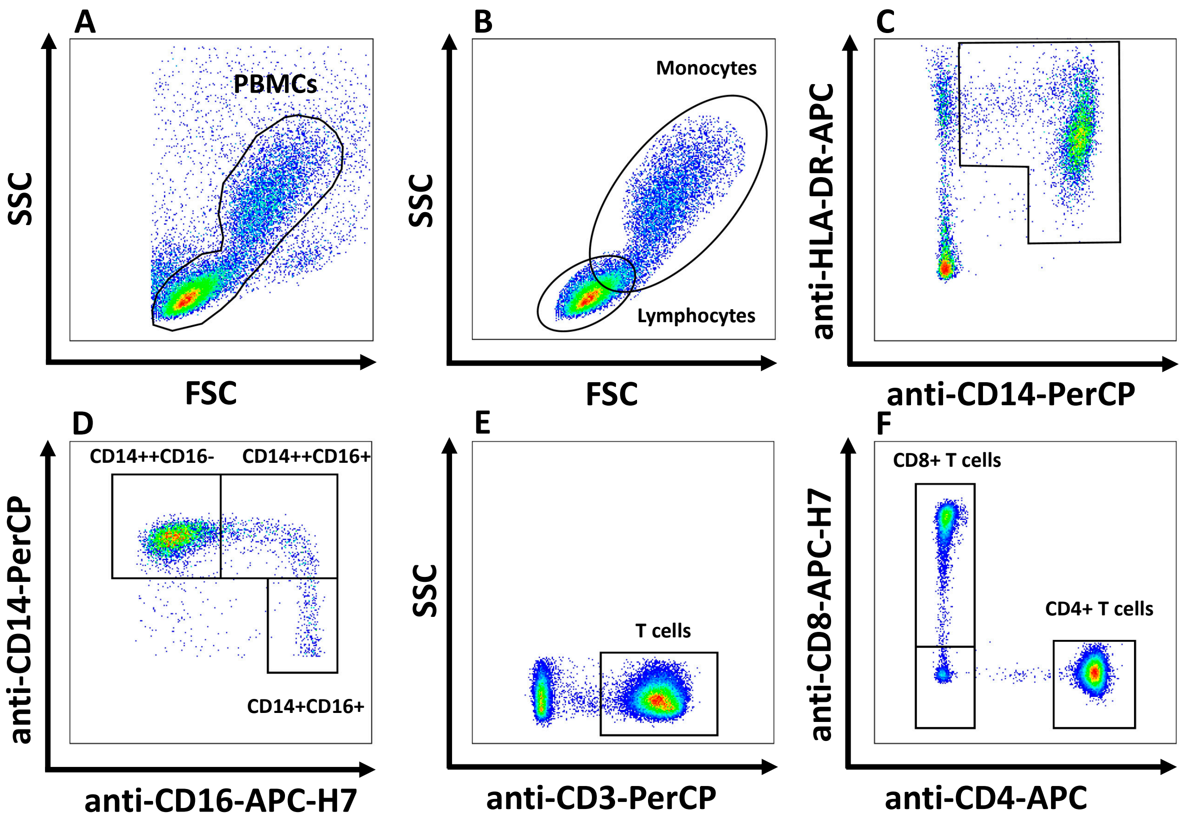

Fig S2. Box plot of BR1 (A) and BR (2) expression on CD3+ (left panel) CD4+ (center panel) and CD8+ (right panel) cells.

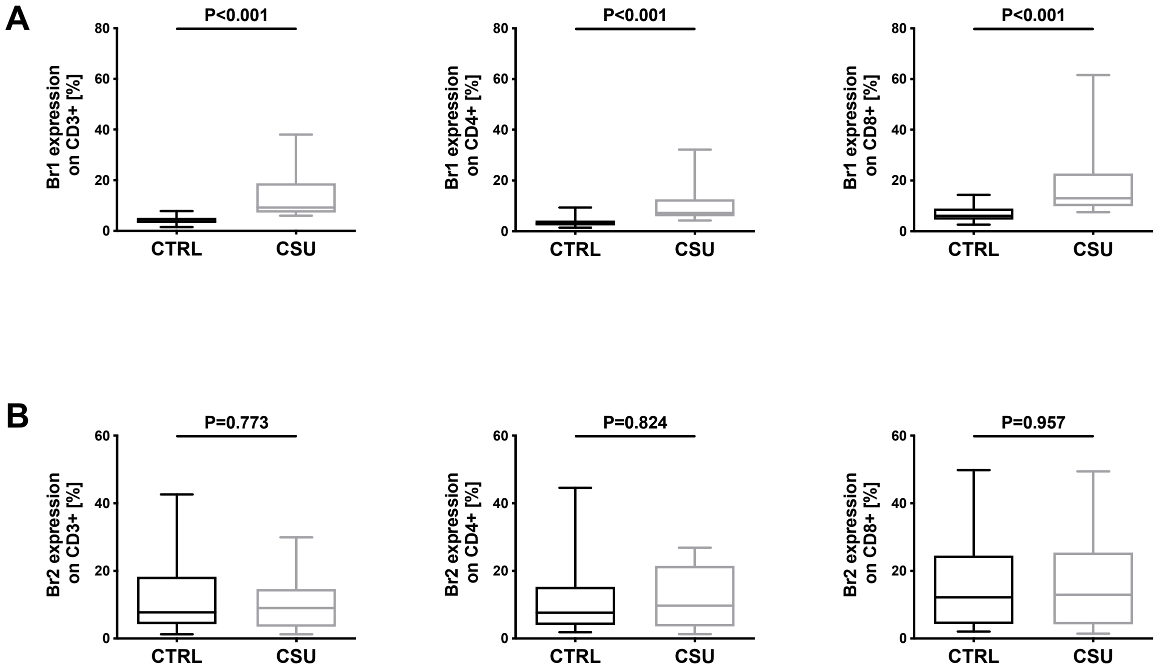

Fig S3. Box plot of BR1 (A) and BR (2) expression on Monocytes, $CD14^{++}CD16^{-}$, $CD14^{++}CD16^{+}$, and $CD14^{+}CD16^{+}$ cells.

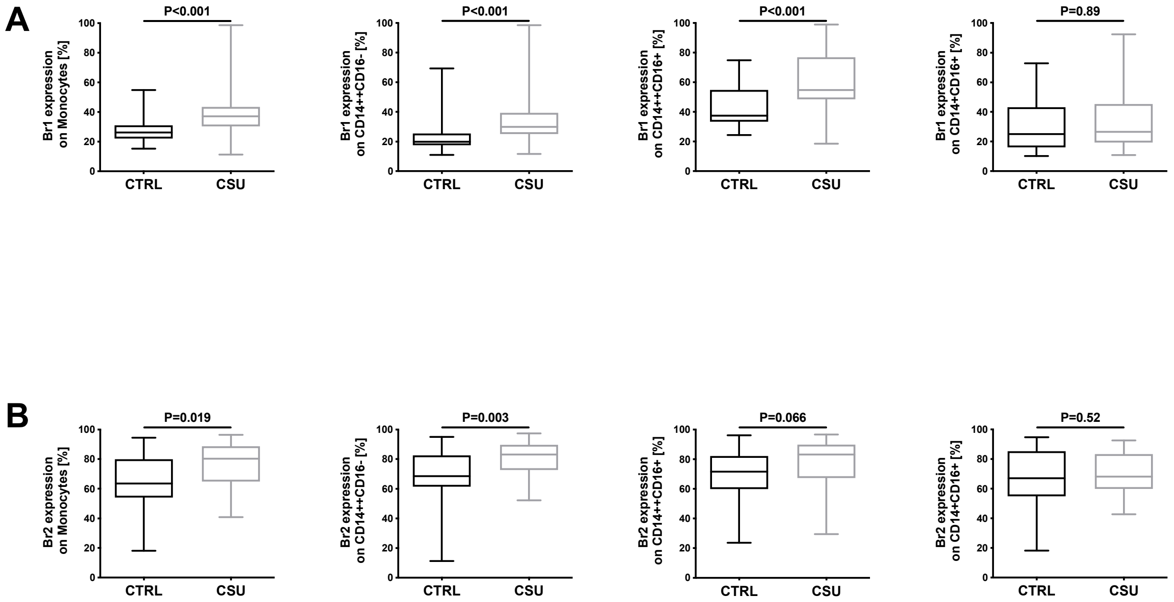

Fig S4. Representative Facs plots for BR1 and BR2 expression on T cells (FMO Control).

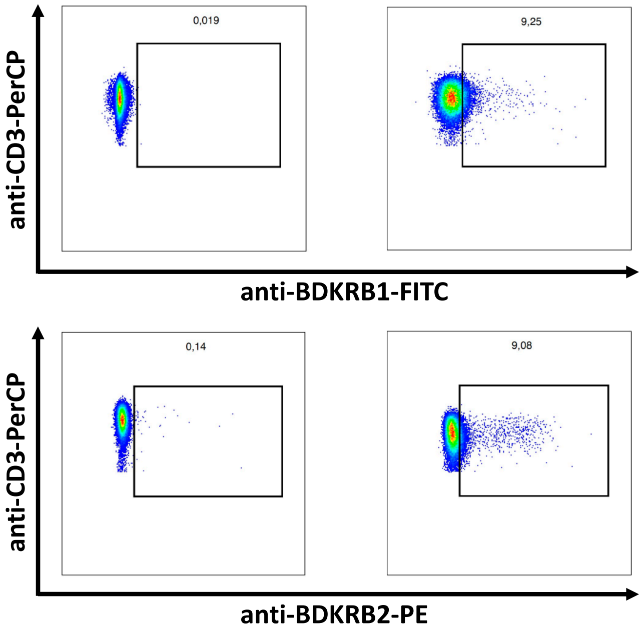

Fig S5. Representative Facs plots for BR1 and BR2 expression on monocytes (FMO Control).

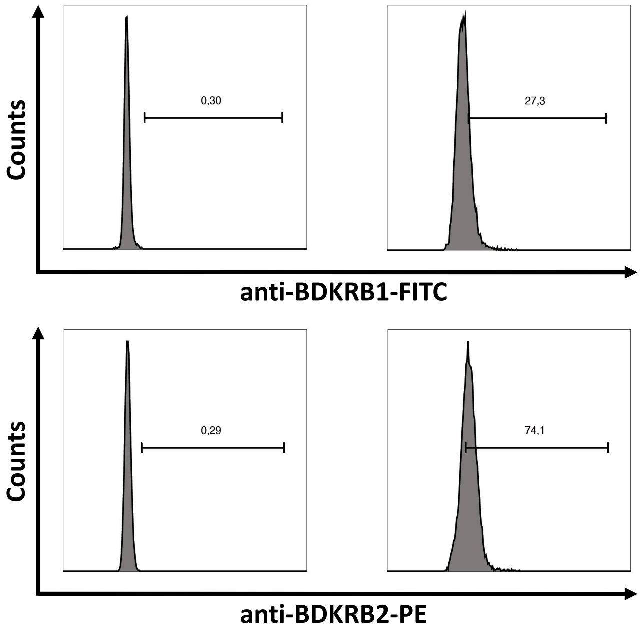

Table S1

Antibody	Clone	Fluorochrome	Manufacturer
anti-CD3	SK7	PerCP	BD, San Jose, CA, USA
anti-CD4	RPA-T4	APC	BD
anti-CD8	SK1	APC-H7	BD
anti-HLA-DR	L243	APC	BD
anti-CD14	MoP9	PerCP	BD
anti–CD16	3G8	APC-H7	BD
anti-BDKRB1	polyclonal	FITC	Bioss Antibodies, Woburn, MA, USA
anti-BDKRB2 (primary)	EPR5646		Abcam, Cambridge, UK
secondary antibody anti-rabbit IgG	2A9	PE	Abcam

Table S1: List of the antibodies used for the staining.
